# Colonic Microbiota and Metabolites Response to Different Dietary Protein Sources in a Piglet Model

**DOI:** 10.3389/fnut.2019.00151

**Published:** 2019-09-24

**Authors:** Rui Li, Ling Chang, Gaifeng Hou, Zehe Song, Zhiyong Fan, Xi He, De-Xing Hou

**Affiliations:** ^1^College of Animal Science and Technology, Hunan Agricultural University, Changsha, China; ^2^Hunan Co-Innovation Center of Animal Production Safety, Changsha, China; ^3^Department of Food Science and Biotechnology, Faculty of Agriculture, Kagoshima University, Kagoshima, Japan

**Keywords:** protein sources, colon, semi-synthetic diet, bacterial communities, metabolites, piglets

## Abstract

Dietary protein sources have the potential to affect the colon microbiome of piglets that will subsequently have a large impact on metabolic capabilities and hindgut health. This study explored the effects of different protein sources on the growth performance, diarrhea rate, apparent ileal digestibility (AID) of crude protein (CP), colonic mucin chemotypes, colonic microbiome, and microbial metabolites of piglets. Twenty-four piglets were randomly divided into four groups that received isoenergetic and isonitrogenous diets containing either Palbio 50 RD (P50), Soyppt-50% (S50), concentrated degossypolized cottonseed protein (CDCP), or fish meal (FM) as the sole protein source. The experimental diets did not affect the estimated daily gain (EDG), but P50 increased fecal score compared with S50 and CDCP. CDCP increased, but P50 reduced AID of CP in comparison to FM and S50. S50 and CDCP increased the amount of mixed neutral-acidic mucins relative to P50. Venn analysis identified unique OTUs in the P50 (13), CDCP (74), FM (39), and S50 (31) groups. The protein sources did not change the colonic bacterial richness or diversity. High *Escherichia* abundance in the P50 and FM, great abundant of *Lactobacillus* in the CDCP, and high *Gemmiger* abundance in the S50 were found. The CDCP tended to elevate valeric acid and branched chain fatty acid (BCFA) concentrations compared with the other diets. The P50 and FM groups had greater ammonia nitrogen and methylamine contents than the S50 and CDCP groups. There was a positive correlation between the *Escherichia* and ammonia nitrogen, the *Lactobacillus* and short chain fatty acid (SCFA), and a negative correlation between the *Gemmige* and BCFA. These findings suggested short-term feeding of different protein sources did not affect the piglets' growth, but P50 increased the diarrhea rate. Potential pathogenic bacteria and detrimental metabolites appeared in the colons of piglets fed P50 and FM, whereas, beneficial effects were conferred upon piglets fed CDCP and S50, thus indicating that available plant proteins (cotton seed, soy) added to the diets of piglets enhanced colon health by reducing protein fermentation.

## Introduction

The weaning of piglets is often accompanied by low growth performance and a high risk of morbidity (mainly diarrhea rate) and death due to immature development of the gut and immune systems, which makes the piglets vulnerable to digestive disorders and highly susceptible to pathogenic bacteria (1–3). Indigestion causes excess undigested nutrients, mainly carbohydrates and protein, to flow into the hindgut where they are fermented by microbes. Carbohydrates fermentation is generally considered beneficial to the intestinal epithelium because it generates short chain fatty acids (SCFAs, acetate, propionate, and butyrate) (4, 5), but protein fermentation is considered to have adverse impacts on intestinal health because it can produce potentially toxic metabolites such as ammonia, biogenic amines, hydrogen sulfide, indols, and phenolic compounds, which are closely associated with the proliferation of pathogenic bacteria and diarrhea (1, 5, 6). Excessive protein fermentation not only wastes protein sources, but also induces some enteric diseases. The inclusion of high-quality of protein (milk by-products, animal proteins, processed proteins, etc.) (1, 2), the reduction of dietary protein intake or protein level (2, 3, 7), the addition of dietary fiber (8–10), and the use of various feed additives (1, 8) are commonly seen in swine production. These nutritional strategies can effectively reduce the outflow of undigested protein into the hindgut and decrease substrates for protein fermentation.

The colonic microbiota plays an important role in digestive physiology and makes a significant contribution to homeostasis (11, 12). Colonic bacteria can catabolize mostly amino acids via the abundant proteases and peptidases produced during fermentation (13). The ileal digestibility of protein and amino acids varies amongst protein source and determines the outflow of substrates into the hindgut for protein fermentation and specific biomarkers (2, 8). Therefore, identification of the biomarkers of various protein sources during fermentation is useful for the selection of suitable protein ingredients for piglets' diets and to provide a reference for dietary modulation to maintain piglets' gut health.

Although enzyme-treated soybean meal (14), cottonseed protein (15), dried porcine solubles (16, 17), and fish meal (18) are widely used in piglets' diets, few studies have evaluated the microbial mechanism of these protein sources on protein fermentation in the hindgut. Therefore, this study investigated the effects of P50, S50, CDCP, and FM on the growth performance, AID of CP, colonic mucin chemotypes, and the composition and metabolites of colonic microbiota in piglets. The results provide a reference for protein sources selection in piglet diets based on hindgut micro-ecosystem regulation.

## Materials and Methods

The Hunan Agricultural University Animal Ethics Committee (Changsha, China) reviewed and approved all experimental protocols. The four protein sources used in the study were Palbio 50 RD (P50, a dried porcine solubles; Bioiberica, S.A, Spain), Soyppt-50% (S50, a enzyme-treated soybean meal; Jiangsu Peptid Biological Co., Ltd, Jiangsu, China), Concentrated degossypolized cottonseed protein (CDCP, a novel processed cottonseed meal; Xinrui Biotech, Hunan, China), and fish meal (FM, steam dried fish meal; Tecnológica de Alimentos S.A., Peru).

### Animal, Diets, and Experimental Design

**Figure 1:**
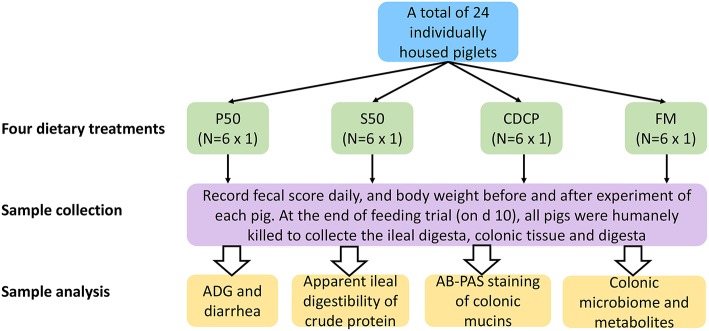
Flow diagram of the experimental design. P50, Palbio 50 RD, a dried porcine solubles; S50, Soyppt-50%, a enzyme-treated soybean meal; CDCP, concentrated degossypolized cottonseed protein; FM, fish meal.

Twenty-four barrows (Landrace × Yorkshire; initial BW = 12.61 ± 1.45 kg) were individually housed in stainless steel metabolism chambers (0.7 × 1.4 m) equipped with a nipple drinker, a feeder, and a fully slatted plastic floor. The piglets were randomly allotted to 1 of 4 diets with 6 piglets in each group (Figure 1). Four cornstarch-based diets were formulated with P50, S50, CDCP, or fish meal as the sole protein (Table 1). Piglets were fed the experimental diets for 9 days, and each piglet was fed a daily level of 5% of the average initial BW of 24 piglets. Feed was provided three times a day, with the daily allowances averaged over the three meals. All piglets had *ad libitum* access to water. On the day 10, piglets were humanly slaughtered to collect the ileal and colonic digesta, and colon tissue.

**Table 1:** Diet composition and nutrient levels (as-fed basis, %).

**Item**	**Diets**^a^			
	**S50**	**CDCP**	**P50**	**FM**
**Ingredient (%)**				
Soyppt-50%	35.00			
Cottonseed protein		29.00		
Palbio 50 RD			34.00	
Fish meal				26.00
Soybean oil		3.00	1.00	3.00
Monocalcium phosphate	1.00	1.00	1.00	1.00
Limestone	2.00	2.00	2.00	2.00
Sucrose	20.00	20.00	20.00	20.00
Titanium dioxide	0.30	0.30	0.30	0.30
Cornstarch	41.00	44.00	41.00	47.00
Sodium chloride	0.40	0.40	0.40	0.40
Vitamin and mineral premix^b^	0.30	0.30	0.30	0.30
Total	100.00	100.00	100.00	100.00
**Calculated nutrient level**				
Crude protein (%)	17.39	17.37	17.30	17.36
Digestible energy (MJ/kg)	15.79	15.74	15.72	15.76
Total starch (%)^c^	48.61	48.23	50.14	47.91

a *S50, Soyppt-50%, a enzyme-treated soybean meal; CDCP, concentrated degossypolized cottonseed protein; P50, Palbio 50 RD, a dried porcine solubles; FM, fish meal*.

b *Provided per kg of diet: 12, 000 IU Vitamin A, 2,400 IU Vitamin D3, 45 IU Vitamin E, 3.0 mg Vitamin K, 0.40 mg Vitamin B1, 6.4 mg Vitamin B2, 0.3 mg Vitamin B6, 36 μg Vitamin B12, 2 mg folic acid, 40 mg nicotinic acid, 20 mg D-pantothenic acid, 0.45 mg biotin, 120 mg Fe, 6 mg Cu, 40 mg Mn, 100 mg Zn, 0.40 mg I, 0.30 mg Se*.

c *Total starch was analyzed using a commercial starch assay kit (Megazyme, Ireland) according to the method 7613.01 of American Association of Cereal Chemists (19)*.

### Sample Collection and Preparation

Body weight per piglet was measured at the beginning and conclusion of the 9-day experimental period to calculate the estimated daily gain (EDG). The fecal score for each piglet was recorded daily, according to the method introduced by Hu et al. (20) (hard feces = 1; slightly soft = 2; soft and partially formed feces = 3; loose, semi-liquid = 4; watery feces = 5). After the feeding trial, all piglets were anesthetized with serazine hydrochloride (Jilin Huamu Animal Health Product Co., Ltd., Changchun, China) at a dosage of 0.5 ml/kg BW by intramuscular injection and humanely euthanatized. The digesta in ileal segment 15 cm prior to ileocecal junction was collected and lyophilized for the determination of apparent ileal digestibility (AID) of crude protein (CP). The digesta in the colonic lumen was collected and stored at −80°C for microbiology and metabolites analyses. The colonic tissue was fixed in Bouin's solution for Histomorphological analysis.

### Measurement of AID of CP

The methods for determining CP and titanium (Ti) contents in diets and lyophilized ileal digesta were detailedly described by Li et al. (21). The AID of CP in diets was calculated as following: AID (%) = [1–(CP_i_ × Ti_d_)/(CP_d_/Ti_i_)] × 100, where CP_i_ and Ti_i_ are the contents of CP and TiO_2_, respectively, in the ileal digesta and CP_d_ and Ti_d_ represent the concentrations of CP and TiO_2_, respectively, in diets.

### AB-PAS Staining of Mucins in Colonic Tissue

To characterize the neutral and acidic mucins in colonic goblet cells, an Alcian blue (pH 2.5)-periodic acid Schiff (AB-PAS) staining procedure, based on the method of Liu et al. (22) and Henwood (23) was conducted. Briefly, the colon tissue samples were fixed in Bouin's solution and then dehydrated and embedded in paraffin blocks. A 5 μm section was sliced, deparaffinized, hydrated, and then stained with AB-PAS. The neutral and acidic mucins in goblet cells were stained in magenta and blue colors, respectively. The results were visualized by computer-assisted microscopy (DT2000, Nanjing dongtu digital technology co. LTD, China) and image-analysis software (Motic Images Plus 2.0, Dongguan, China).

### Analysis of SCFA, BCFA, and Nitrogen Metabolites

Approximately 1.0 g of colon digesta was diluted with 10 mL of ultra-pure water, vortexed for 2 min, centrifuged at 10,000 × g for 10 min and filtered (0.45 μm) to collect the filtrate. A 2-mL portion of the filtrate was transferred to a 10-ml centrifuge tube, and 0.2 mL of 50% sulfuric acid and 2 mL of diethyl ether were added successively. The solution was vortexed for 2 min, centrifuged at 10,000 × g for 5 min and placed at 4°C for 30 min. The supernatant was analyzed for SCFA (acetic acid, propionate acid and butyric acid) and BCFA (isobutyric acid, isovaleric acid and valeric acid) using an Agilent 7890A gas chromatographer (Agilent Technologies Inc., Palo Alto, CA) equipped with an HP-FFAP elastic quartz capillary vessel column (30 m × 0.25 mm × 0.25 μm). The carrier gas was high-purity nitrogen (99.999%) with a constant flow rate of 2.0 mL/min. The column temperature procedure was from 100°C for 1 min then increasing by 5°C/min to reach 150°C for 5 min. The inlet and detector (FID) temperatures were 270°C and 280°C, respectively. The inlet volume was 2.0 μL, as measured by splitless injection.

The contents of ammonia nitrogen (NH_3_-N) in samples were determined using Nessler's reagent spectrophotometry as described by Chen et al. (24). Briefly, 0.5 g of colonic digesta was mixed with 5 mL of ammonia-free water and centrifuged at 5,000 × g for 15 min at 4°C to collect the supernatants. A 1-mL portion of supernatants was transferred to a 50-mL aseptic tube filled with 19 mL of ammonia-free water and 1 mL of potassium sodium tartrate, after which 1.5 mL of Nessler's reagent was added and the mixture was left to stand for 10 min. The OD value of the mixture was measured at 420 nm against ammonia-free water on a multi-scan spectrum microplate spectrophotometer (Thermo Fisher Scientific (China), Co., Ltd, Shanghai, China).

The concentrations of biogenic amines in the colonic digesta, mainly methylamine, cadaverine, putrescine, histamine, and spermidine, were analyzed with high- performance liquid chromatography (HPLC) according to the method introduced by Fan et al. (25). In short, 0.2 g of colonic digesta was weighed into a 2-mL tube, and 1 mL of trichloroacetic acid (TCA) was added and homogenized for 10 min. Afterward, the mixture was centrifuged at 3,600 × g for 10 min at 4°C to collect the supernatants. An equal volume of n-hexane was mixed with the supernatants and vortexed for 5 min, after which the organic phase was removed and the aqueous phase was re-extracted in the same way. The extracts were transferred into a 50-mL tube, and 20 mL of internal standard, 1.5 mL of saturated sodium bicarbonate (NaHCO_3_), 1 mL of dansyl chloride, and 1 mL of sodium hydroxide (NaOH) were then added successively. The mixture was heated at 60°C for 45 min with occasional mild shaking. Next, 100 μL of termination solution (i.e., ammonia) was added to the mixture, and the solution was maintained 40°C in a water bath to vaporize the acetone by blow-drying with N_2_. Finally, the sample was extracted twice with 3 mL of diethyl ether, and the collected extracts were blow-dried with N_2_, and the residue was then re-dissolved in acetonitrile solution for HPLC analysis. An ammonium acetate-acetonitrile gradient elution program was used on an Agilent 1200 equipped with a variable wavelength detector (VWD) and a reversed–phase ZORBAX 80 A Extend–C18 (4.6 mm × 250 mm; 5 μm) column (Agilent Technologies, USA). The flow rate, temperature and wavelength were set at 1.0 mL/min, 30°C and 260 nm, respectively.

### DNA Extraction, PCR Amplification, Library Preparation, and Sequencing

Twenty colonic digesta samples from 4 dietary groups (5 per group) were randomly selected for 16S rRNA sequencing analysis. The total genomic DNA of each sample was extracted by using the TIANamp Stool DNA kit (Tiangen Biotech (Beijing) Co., Ltd, China) according to the manufacturer's instructions. The quantity and quality of extracted DNAs were measured using a NanoDrop ND-1000 spectrophotometer (Thermo Fisher Scientific, USA) and agarose gel electrophoresis, respectively. Nineteen acceptable DNA samples (one low-quality DNA sample from the FM group was discarded) were sent to BGI (Wuhan, China) for 16S rRNA sequencing.

The V3-V4 hypervariable region of the bacterial 16S rRNA gene was amplified with the barcoded universal primers (341F-806R). All the PCR reactions were performed in 30-μL reaction volumes containing 1.0 μL of each primer, 1.0 μL of DNA template, 15 μL of Phusion® PCR Master Mix (New England Biolabs, USA), and 12 μL of sterile water. After the PCR amplification ended under the set cycle condition, agarose gel electrophoresis and a GeneJET Gel Extraction Kit (Thermo Fisher Scientific, USA) were used to determine, and extract and purify the PCR products. Sequencing libraries were prepared using an NEB Next® Ultra^TM^ DNA Library Prep Kit for Illumina (New England Biolabs, USA) according to the manufacturer's methods. Sequencing was conducted on the Illumina HiSeq platform by HiSeq2500 PE250 (Illumina, USA), and 250 bp paired-end reads were generated.

### Bioinformatics Analysis

Paired-end reads from the sequencing data were screened and processed by FLASH (v1.2.11) (26, 27) and QIIME (v1.8.0) (28). Chimeric sequences produced by the PCR amplification were removed by UCHIME (v4.2.40) (29). The assembled tags were clustered as OTU via USEARCH (V7.0.1090) (30). Sequences with 97% similarity were assigned to the same operational taxonomic units (OTUs), selected by UPARSE. Meanwhile, we picked a representative sequence for each OTUs and used the RDP (v2.2) classification to assign taxonomic data to each representative sequence, with a confidence threshold of 0.6. Taxonomy classifications were set against Greengene (V201305) (31) and RDP (Release9 201203) (32). An OTU table was further generated to record the abundance of each OTU in each sample, and a profiling histogram was made using R software (v3.1.1) to represent the relative abundance of taxonomic groups from phylum to species. A Venn diagram was generated to visualize the occurrence of shared and unique OTUs among groups^1^ Alpha diversity indices based on the OTUs in all samples were analyzed with mothur (v1.31.2) according to the calculation formula of each index^2^ and displayed with Prism software (v7.0). Beta diversity was evaluated using the Unifrac metric, and the UPGMA tree based on the unweighted UniFrac distances between the OTUs was displayed by QIIME (v1.80). Principal component analysis (PCA) was also conducted with R software (v3.1.1) based on the OTUs among samples from different groups. A heatmap was created using R software (v3.1.1) after performing the logarithmic transformation of relative abundance in each sample. LefSe analysis was conducted based on the relative abundance of annotated taxonomic composition to identify biomarkers for microbial communities from phylum to genus^3^.

### Statistical Analysis

EDG, fecal score, and colonic SCFAs, ammonia nitrogen, biogenic amines and alpha diversity indexes were analyzed by One-Way ANOVA using the GLM procedure of SPSS 17.0 (SPSS Inc., Chicago, IL, USA), with individual piglet as an experimental unit. Unweighted Unifrac distances were compared based on approximately-maximum-likelihood to construce UPGMA trees. The Kruskal test was used for *post hoc* comparison of taxonomy. For all tests, *P* < 0.05 was considered as significant difference, while 0.05 < *P* < 0.10 as a tendency. Correction between metabolites and microbiota in colonic digesta was done with Spearman's rank- order correlation test in R software (v3.1.1).

## Results

### Growth Performance, Fecal Scores, and AID of CP

**Figure 2:**
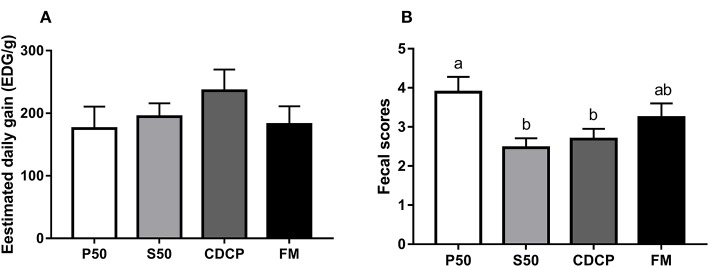
The estimated daily gain (EDG, **A**) and fecal score **(B)** of piglets fed different dietary protein sources. P50, Palbio 50 RD, a dried porcine solubles; S50, Soyppt-50%, an enzyme-treated soybean meal; CDCP, concentrated degossypolized cottonseed protein; FM, fish meal. Different letters above the error bar mean significant difference (*P* < 0.05).

No differences in EDG were observed among the four dietary groups (*P* = 0.173) (Figure 2). However, the piglets in the P50 group elevated fecal scores compared with those in the S50 and CDCP groups (*P* = 0.039). Protein source significantly affected the AID of CP in diets (*P* < 0.01) (published in our previous paper). CDCP had the highest AID of CP and P50 had the lowest AID of CP. No differences in AID of CP were found between S50 and FM (*P* > 0.05).

### Characterization of Mucin Chemotypes

**Figure 3:**
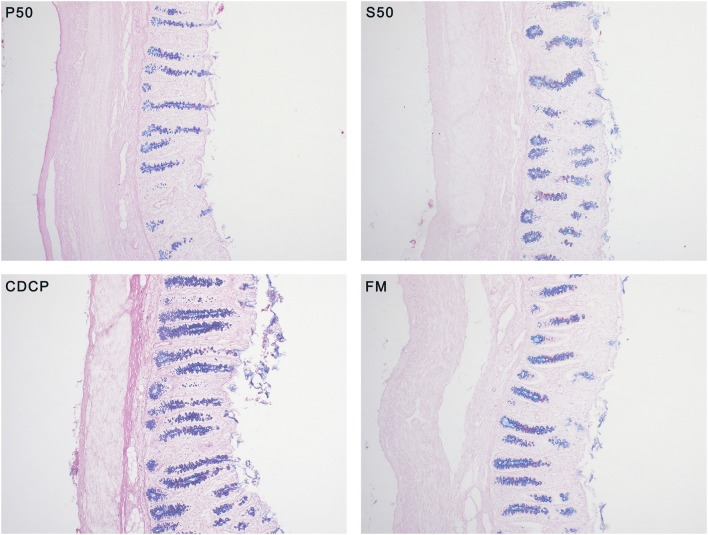
Alcian blue (pH 2.5)-periodic acid Schiff (AB-PAS) stained section (100×) in colon tissues. Neutral mucins are marked in magenta, while acidic mucins in blue. P50, Palbio 50 RD, a dried porcine solubles; S50, Soyppt-50%, a enzyme-treated soybean meal; CDCP, concentrated degossypolized cottonseed protein; FM, fish meal.

With AB-PAS staining, the amount of mixed neutral-acidic mucins in colon tissue of piglets fed S50 and CDCP diets were increased (*P* < 0.05) compared with those fed P50 (Figure 3).

### Microbial Community Structure in Colonic Digesta

**Figure 4:**
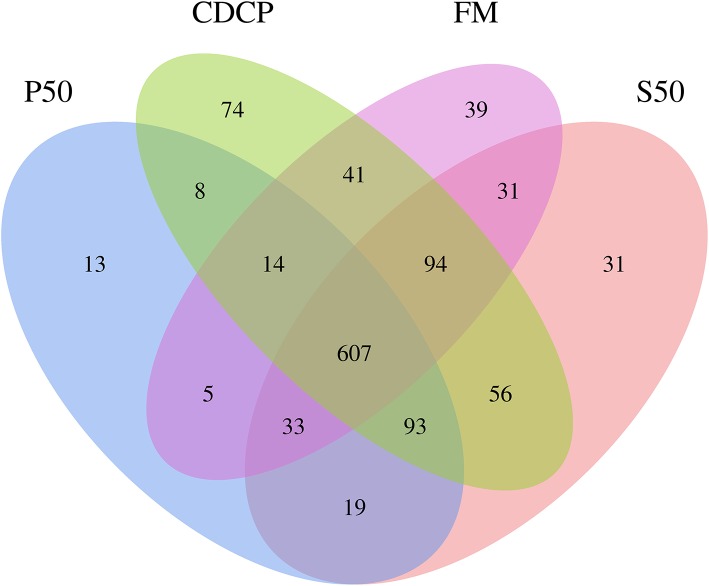
Venn pictures exhibit shared and the unique OTUS among dietary treatments. P50, Palbio 50 RD, a dried porcine solubles; S50, Soyppt-50%, a enzyme-treated soybean meal; CDCP, concentrated degossypolized cottonseed protein; FM, fish meal.

**Figure 5:**
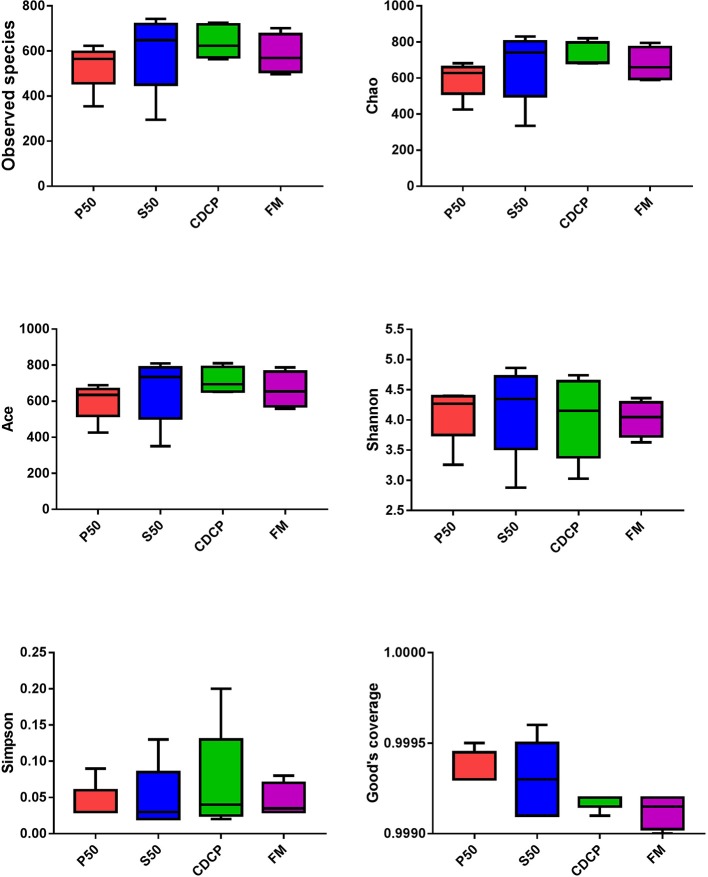
Difference analysis of alpha diversity index among different dietary treatments. P50, Palbio 50 RD, a dried porcine solubles; S50, Soyppt-50%, a enzyme-treated soybean meal; CDCP, concentrated degossypolized cottonseed protein; FM, fish meal.

**Figure 6:**
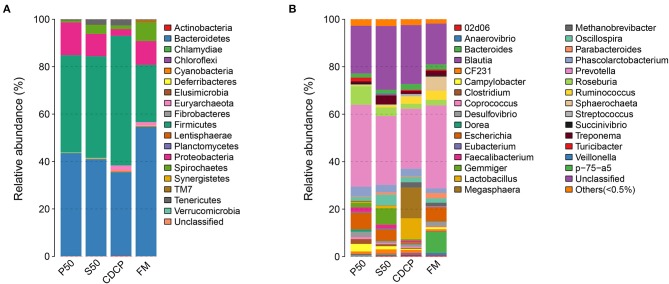
Relative abundance of colonic bacteria, at phylum **(A)** and genus **(B)** level, in piglets fed diets containing P50, S50, CDCP, and FM. Representative sequences for each OUT were used to annotate taxonomic information. P50, Palbio 50 RD, a dried porcine solubles; S50, Soyppt-50%, a enzyme-treated soybean meal; CDCP, concentrated degossypolized cottonseed protein; FM, fish meal.

The Venn analysis identified 13, 74, 39, and 31 unique OTUs in the P50, CDCP, FM, and S50 dietary groups, respectively (Figure 4). Dietary protein sources did not affect the alpha diversity indices (Figure 5). *Firmicutes, Bacteroidetes, Proteobacteria*, and *Spirochaetes* were the four most abundant phyla (Figure 6A). The *Firmicutes*/*Bacteroidetes* ratios in CDCP, S50, P50, and FM were 1.54, 1.03, 0.95, and 0.45, respectively. FM had a lower abundance of *Firmicutes* (*P* = 0.025), but a greater abundance of *Spirochaetes* (*P* = 0.040) and TM7 (*P* = 0.007) than the other three groups, S50 and CDCP had increased *Tenericutes* abundance compared with P50 and FM (*P* = 0.012) (Supplementary Table S2). Down to the genus level, P50 and FM had high abundances of *Escherichia* (*P* = 0.029) and *Clostridium* (*P* = 0.041), whereas, CDCP had abundances of *Lactobacillus* (*P* = 0.002) and *Megasphaera* (*P* = 0.033) and S50 had a high abundances of *Gemmiger* (*P* = 0.018) (Figure 6B).

**Figure 7:**
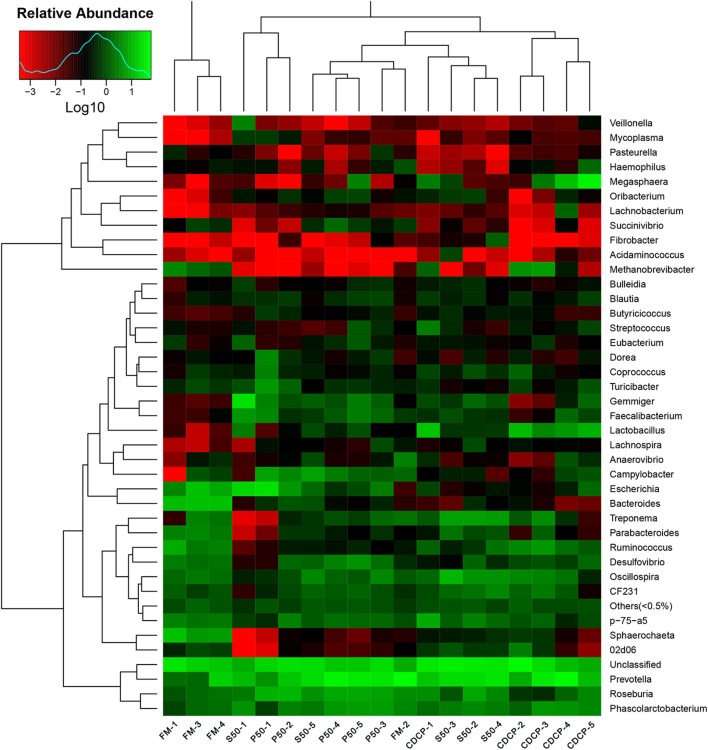
Relative abundance of each taxonomic genus in different dietary treatments. The rows show the top 50 genera in each sample and the color scale illustrates the comparison of each genera among the samples. P50, Palbio 50 RD, a dried porcine solubles; S50, Soyppt-50%, a enzyme-treated soybean meal; CDCP, concentrated degossypolized cottonseed protein; FM, fish meal.

The heatmap results showed that *Phascolarctobacterium, Prevotella*, and *Roseburia* were the most abundant genera within the four dietary groups (Figure 7). CDCP had greater *Lactobacillus* abundance than the other three dietary groups, whilst P50 and FM had increased *Escherichia* abundance compared with CDCP and S50.

**Figure 8:**
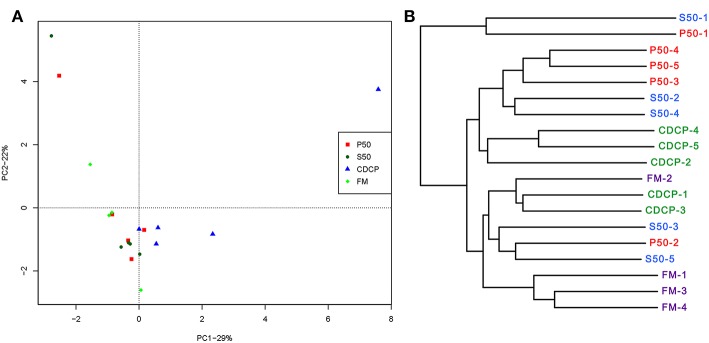
Comparison of colonic microbial structures within piglets fed different dietary protein sources. **(A)** Principal component analysis (PCA) based on OTUs among samples of different groups, each point represents one sample. **(B)** UPGMA tree, all revealing significant differences among groups based on Unweighted UniFrac distances of OTUs community. P50, Palbio 50 RD, a dried porcine solubles; S50, Soyppt-50%, a enzyme-treated soybean meal; CDCP, concentrated degossypolized cottonseed protein; FM, fish meal.

PCA, a multivariate analysis, was used to compare the overall composition of the microbiota in the colonic samples from various dietary groups (Figure 8A). The first two components accounted for 52% of the variation. PC1 mainly represented the diet-induced variations, whilst PC2 explained intra-group variations in microbial structure. Greater variations were seen in P50 and FM than in CDCP and S50, whilst P50, S50, and CDCP had similar microbial communities. The UPGMA tree analysis (Figure 8B) revealed that remarked differences in the structure of colonic microbiota among various dietary groups, suggesting that the protein sources had a notable impact on colonic microbial communities.

**Figure 9:**
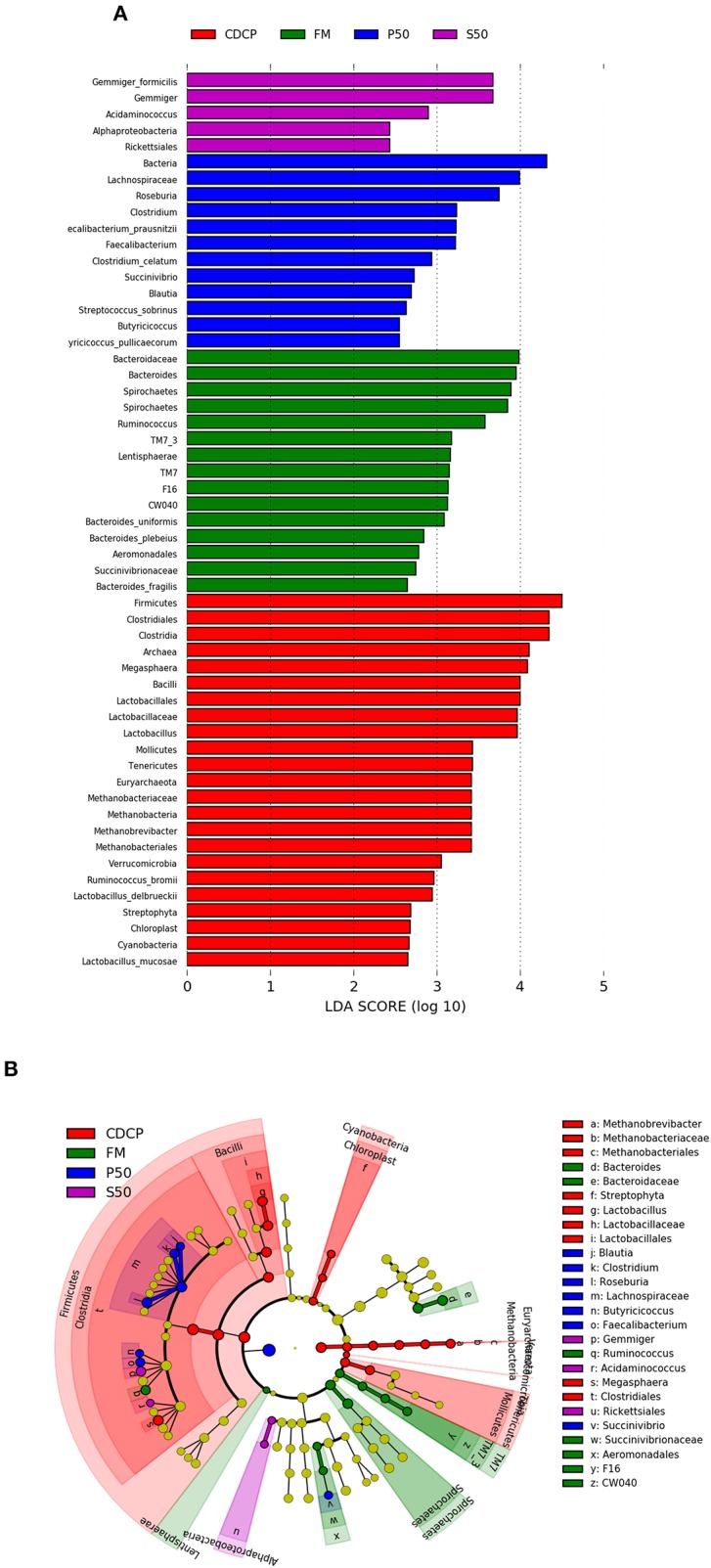
LefSE analysis of colonic microbiota among different dietary groups. **(A)** LDA scores are calculated for characteristics at the OUT level. **(B)** Cladogram shows the relative abundance of OTUs from the phylum to genus level, the node size represented the average relative abundance of the taxon, the yellow node indicated no difference among groups, but the other color nodes suggested the significant difference, the letters identified the taxon name with notable difference among groups. P50, Palbio 50 RD, a dried porcine solubles; S50, Soyppt-50%, a enzyme-treated soybean meal; CDCP, concentrated degossypolized cottonseed protein; FM, fish meal.

LEfSe analysis showed 55 different OTUs among the 4 dietary protein groups, and the abundance of 5 OTUs was low in S50, with 12 in P50, 15 in FM, and 23 in CDCP (Figure 9A). There was a high abundance of *Lactobacillus* in CDCP, *Bacteroides* in FM, *Clostridium* in P50, and *Gemmiger* in S50, respectively (Figure 9B).

### Fermentation Metabolites of Colonic Digesta

CDCP showed a trend of elevated valeric acid (*P* = 0.052) and BCFA (*P* = 0.064) concentrations (Table 2). P50 and FM had greater ammonia nitrogen and methylamine contents than S50 and CDCP (*P* = 0.041) (Table 3).

**Table 2:** Colonic short chain fatty acid (SCFAs) (mg/g).

**Item**	**P50**	**S50**	**CDCP**	**FM**	**P**
Acetic acid	1.89 ± 0.29	2.22 ± 0.36	2.40 ± 0.14	1.67 ± 0.06	0.484
Propionate acid	1.30 ± 0.13	1.76 ± 0.21	1.66 ± 0.10	1.32 ± 0.07	0.260
Butyric acid	0.73 ± 0.08	1.23 ± 0.07	1.17 ± 0.11	0.90 ± 0.03	0.622
SCFA	3.92 ± 0.32	5.20 ± 0.85	5.23 ± 1.05	3.89 ± 0.56	0.414
Isobutyric acid	0.028 ± 0.002	0.076 ± 0.003	0.069 ± 0.005	0.088 ± 0.008	0.295
Isovaleric acid	0.013 ± 0.002	0.062 ± 0.010	0.245 ± 0.007	0.174 ± 0.015	0.278
Valeric acid	0.232 ± 0.002^b^	0.139 ± 0.038^b^	1.144 ± 0.131^a^	0.253 ± 0.002^b^	0.052
BCFA	0.273 ± 0.002^b^	0.277 ± 0.014^b^	1.145 ± 0.028^a^	0.515 ± 0.008^ab^	0.064
Total SCFAs	4.19 ± 0.44	5.47 ± 0.73	6.69 ± 1.01	4.41 ± 0.61	0.253

a,b *means with different superscripts in the same row differ significantly (P <0.05)*.

**Table 3:** Colonic nitrogen metabolites.

**Item**	**P50**	**S50**	**CDCP**	**FM**	**P**
Ammonia nitrogen (mg/mL)	299.89 ± 27.29^a^	242.53 ± 21.33^b^	240.17 ± 10.91^b^	307.24 ± 17.62^a^	0.041
Biogenic amines (μg/g)					
Methylamine	152.00 ± 21.39^a^	80.46 ± 12.35^b^	66.76 ± 4.09^b^	119.13 ± 17.35^a^	0.027
Cadaverine	277.02 ± 45.55	249.62 ± 23.82	237.09 ± 53.16	296.99 ± 60.15	0.414
Putrescine	256.57 ± 73.32	259.73 ± 58.58	177.95 ± 35.04	269.32 ± 65.60	0.295
Histamine	573.14 ± 36.12	648.36 ± 65.00	969.86 ± 92.69	326.38 ± 35.66	0.278
Spermidine	169.15 ± 44.00	247.11 ± 60.73	360.69 ± 57.01	144.21 ± 61.53	0.253

a, b *means with different superscripts in the same row differ significantly (P <0.05)*.

### Correction Between Metabolites and Microbiota in Colonic Digesta

**Figure 10:**
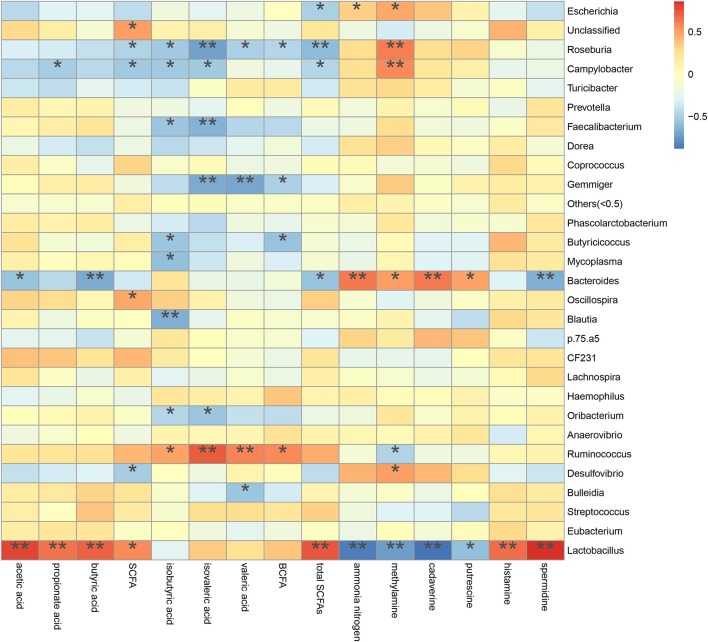
Correlation between metabolite contents and relative microbiota abundance in colonic digesta from piglets. Correlation is indicated by a color gradient from blue to red based on Spearman's correlation coefficients (*P < 0.05; **P < 0.01).

A spearman's rank correlation analysis was done to infer taxa associated with metabolic potentials (Figure 10). We found that the increased *Escherichia* abundance in FM and P50 group was positively correlated with ammonia nitrogen (*P* < 0.05) and methylamine (*P* < 0.05), but negatively corrected with total SCFAs (*P* < 0.05). *Lactobacillus*, contained an increased abundance in CDCP group, was positively correlated with acetic acid (*P* < 0.01), propionate acid (*P* < 0.01), butyric acid (*P* < 0.01), SCFA (*P* < 0.05), total SCFAs (*P* < 0.01), histamine (*P* < 0.01), and spermidine (*P* < 0.01), but negatively corrected with ammonia nitrogen (*P* < 0.01), methylamine (*P* < 0.01), cadaverine (*P* < 0.01), and putrescine (*P* < 0.05). *Gemmiger*, had an increased abundance in S50 group, was negatively corrected with isovaleric acid (*P* < 0.01), valeric acid (*P* < 0.01), and BCFA (*P* < 0.05).

## Discussion

Dietary protein is mainly digested into peptides and amino acids in the foregut via the action of proteases (13, 25). The digestion and absorption of protein is closely associated with the increased lean muscle mass (33). The weight gain of piglets is primary lean muscle mass due to their rapid growth and quick protein turnover (34, 35). In our study, equal amounts of feed with similar DE and CP were fed to each piglet daily to ensure the similar dietary protein and energy intakes. This helps to explain, along with the short experimental period (9 days), why the EDG of the piglets showed no difference among the dietary groups. However, the P50 group increased the diarrhea rate, perhaps because of the high salt content and poor carriers added to co-dried P50, which is the porcine mucosa hydrolysate after heparin extraction and is usually sprayed into a soybean meal carrier (16, 17, 36). Additionally, the reduced AID of CP in the P50 group in our previous study (21) could also explain the increased diarrhea rate, because more undigested protein flow into the hindgut for protein fermentation.

The porcine gastrointestinal tract harbors highly diverse and dynamic microbial communities that play a vital role in maintenance of intestinal health, which contributes to nutrient utilization and modulation of the immune system (5, 37). Diet composition, especially carbohydrates and protein, reflects the substrates available for the intestinal microbiota, which affects their composition and metabolic activity (38). Researchers and the feed industry have paid increasing attention to dietary protein manipulation of the intestinal microbiota in piglets (7, 15, 39, 40). Our experiment determined the colonic microbiome of piglets fed diets containing either P50, S50, CDCP, or FM as sole protein source via 16S rRNA high-throughput sequencing. On average, 164,709 effective tags were achieved for each sample with a high Good's coverage (>99.90%) (Supplementary Table S1). Meanwhile, protein sources did not affect the alpha diversity indices, suggesting that the ecological diversity of the colonic microbiota was quite similar among the samples. The alpha diversity reflects the species diversity in a single sample, including species richness indices (observed species, chao, and ace), species diversity (Shannon, Simpson, and Good's coverage) (31, 41). Our findings concurred with those of Cao et al. (15), who found no change in alpha diversity of piglets fed diets containing soybean meal, cottonseed meal, SBM and CSM or fish meal. However, Venn analysis identified 13, 74, 39, and 31 unique OTUs in the P50, CDCP, FM, and S50 groups, respectively, indicating that specific bacteria appeared in piglets fed different diets.

At the phylum level, *Firmicutes, Bacteroidetes, Proteobacteria*, and *Spirochaetes* were the major phyla in colonic digesta (Figure 6A), similar to the findings of a study by Cao et al. (15). The value for *Firmicutes*/*Bacteroidetes* ratio in CDCP, S50, P50, and FM was gradually reduced (Figure 7). This index is positively correlated with obesity (42), and interestingly, no differences in EDG were found among the four groups (Figure 2A). Down to the genus level, there were high abundances of *Escherichia* and *Clostridium* in P50 and FM, of *Lactobacillus* and *Megasphaera* in CDCP, and of *Gemmiger* in S50 (Figure 6B). These observations were also confirmed by heatmap (Figure 7) and LEfSe analysis (Figure 9), which can identify unique biomarkers for high dimensional gut bacteria composition (43). *Prevotella* is a dominant genus in all dietary groups (Figure 7), and the bacteria in this genus mainly used carbohydrates as substrates (44); thus, our finding might be due to the similar total starch content among diets (Table 1). *Bacteroides, Coliform* bacteria, and *Clostridium* are closely related to protein fermentation and often increase the risk of diarrhea (2). The high abundance of *Escherichia* and *Clostridium* in P50 and FM can explain the increased fecal scores. The finding was similar to that of Heo et al. (45), who reported that the occurrence of pathogenic diarrhea in piglets was mainly due to the release of endotoxin by *Escherichia coli*, which adhered to gut epithelial cells. PCA and UPGMA tree revealed greater variations were seen in P50 and FM than in CDCP and S50, suggesting that the intestinal microbiota responds differently to animal protein.

SCFA (acetate, propionate, and butyrate) and BCFA (isobutyrate, isovalerate, and valerate) are generated from fermentation of carbohydrates and protein in the colon (1). Both carbohydrates and protein fermentation produce SCFA, whilst BCFA are coming from deamination of valine, leucine, and isoleucine (25). In piglets, considerable amounts of carbohydrates are fermented in the proximal colon, but protein fermentation takes place more distally, especially when there is a shortage of available fermentable carbohydrates (1). Our results (Table 2) showed that dietary protein did not affect SCFAs profiles, but the CDCP group showed a tendency of increased valeric acid and BCFA contents. Some reports have indicated that BCFA play a role in maintaining intestinal cell integrity (46, 47) and suggested that CDCP might ameliorate the colon health. This was consistent with the study by Cao et al. (15), who found a beneficial effect of cottonseed meal on the gut of piglets. However, Rist et al. (1) suggested that BCFA might be an indicators of the extent of protein fermentation and exerted detrimental effects upon the host.

The decarboxylation and deamination of amino acids generate bioamines and ammonia, respectively. Ammonia, amines, phenols, and indoles are considered as potentially toxic products, which may interfere with epithelial cell turnover and inhibit pig growth (13, 24). In this study, S50 and CDCP had lower ammonia nitrogen and methylamine contents than P50 and FM (Table 3), possibly because plant protein (S50 and CDCP) contains dietary fiber, which help to reduce protein fermentation. Many researchers have suggested that the addition of dietary fiber to piglets' diets could reduced protein fermentation and diarrhea rate (6, 8, 9, 37, 48). The finding that CDCP and S50 decreased *Escherichia* abundance compared with P50 and FM (Figure 7) could also explain the reduced nitrogen metabolites. Additionally, the increased *Escherichia* abundance in FM and P50 was positively correlated with ammonia nitrogen and methylamine, but negatively corrected with total SCFAs (Figure 10), which could also provide an evidence for the beneficial effect of CDCP and S50 to colon.

Mucin layer spreads over the surface of mucosa and safeguards the intestine and its structural integrity (22). Mucins is broadly categorized into neutral and acidic chemotypes. In our study, we distinguished and detected the two types of mucins and we found that CDCP and S50 increased the amount of mixed neutral-acidic mucins in colon, which might ascribe the reduced protein fermentation in colon of piglets fed plant protein sources (Figure 3).

## Conclusion

Protein intake from 4 different diets (P50, S50, CDCP, and FM) had similar effects on piglet growth, but P50 increased diarrhea rate during short-term feeding. CDCP and S50 reduced the protein flows into hindgut and increased the amount of mixed neutral-acidic mucins in colon. Potential pathogenic bacteria (*Escherichia*) and detrimental metabolites (ammonia) were found in the colons of piglets fed P50 and FM, whilst beneficial effects (*Lactobacillus* and BCFA) were seen in pigs fed CDCP. These findings indicate that the addition of available plant protein such as CDCP to the diet of piglets enhances colon health by reducing protein fermentation.

## Data Availability

The datasets for this manuscript are not publicly available because we have not uploaded the datasets to the NCBI due to some secret data, temporarily. Requests to access the datasets should be directed to lirui181000@163.com.

## Ethics Statement

The Hunan Agricultural University Animal Ethics Committee (Changsha, China) reviewed and approved all experimental protocols.

## Author Contributions

RL, XH, and D-XH design the experiment and revised the manuscript. RL, ZS, and GH conducted the experiments. RL, ZS, ZF, and XH offered the experimental reagents and materials. RL analyzed the data and finished the manuscript. RL, LC, GH, ZS, and D-XH prepared the figures and edited the manuscript. All authors reviewed the manuscript.

### Conflict of Interest Statement

The authors declare that the research was conducted in the absence of any commercial or financial relationships that could be construed as a potential conflict of interest.

## Footnotes

^1^http://www.ehbio.com/ImageGP/index.php/Home/Index/VennDiagram.html

^2^http://www.mothur.org/wiki/Calculators

^3^http://huttenhower.sph.harvard.edu/galaxy/
