# Supplementary material for: Colonic Microbiota and Metabolites Response to Different Dietary Protein Sources in a Piglet Model
Source: Front Nutr. 2019 Sep 24;6:151. doi: 10.3389/fnut.2019.00151 (PMC6768948; doi:10.3389/fnut.2019.00151)
Supplement: Supplementary file 1 [file Table_1.DOC]

**Supplementary Infromation**

**Supplementary table S1.** Raw reads and selected effective sequences in each sample.

| Sample  Name | Raw  Reads | Clean  Reads | Read  Utilization  Ratio(%) | Total Pairs Read  Number | Connect Tag  Number | Connect  Ratio(%) | Average Length  And SD | Tag number | OUT number |
| --- | --- | --- | --- | --- | --- | --- | --- | --- | --- |
| P50-1 | 171204*2 | 166977*2 | 97.53 | 166977 | 166823 | 99.91 | 252/0 | 123749 | 354 |
| P50-2 | 173464*2 | 168484*2 | 97.13 | 168484 | 168227 | 99.85 | 252/1 | 121625 | 565 |
| P50-3 | 169819*2 | 164946*2 | 97.13 | 164946 | 164728 | 99.87 | 252/0 | 117962 | 623 |
| P50-4 | 173969*2 | 168234*2 | 96.70 | 168234 | 167939 | 99.82 | 252/0 | 131521 | 560 |
| P50-5 | 172591*2 | 167356*2 | 96.97 | 167356 | 166965 | 99.77 | 252/0 | 120483 | 569 |
| S50-1 | 171881*2 | 167020*2 | 97.17 | 167020 | 166780 | 99.86 | 252/0 | 131986 | 295 |
| S50-2 | 171402*2 | 165717*2 | 96.68 | 165717 | 165434 | 99.83 | 252/0 | 115131 | 742 |
| S50-3 | 172070*2 | 166371*2 | 96.69 | 166371 | 166061 | 99.81 | 252/1 | 123379 | 606 |
| S50-4 | 172874*2 | 165973*2 | 96.01 | 165973 | 165637 | 99.8 | 252/0 | 126293 | 697 |
| S50-5 | 173443*2 | 167846*2 | 96.77 | 167846 | 167409 | 99.74 | 252/0 | 118561 | 648 |
| CDCP-1 | 172040*2 | 166020*2 | 96.50 | 166020 | 165682 | 99.8 | 252/0 | 127117 | 725 |
| CDCP-2 | 171549*2 | 165478*2 | 96.46 | 165478 | 165234 | 99.85 | 252/1 | 110361 | 712 |
| CDCP-3 | 171989*2 | 165329*2 | 96.13 | 165329 | 164972 | 99.78 | 252/0 | 111853 | 623 |
| CDCP-4 | 171866*2 | 166175*2 | 96.69 | 166175 | 165863 | 99.81 | 252/0 | 126485 | 579 |
| CDCP-5 | 171410*2 | 165690*2 | 96.66 | 165690 | 165446 | 99.85 | 252/0 | 126389 | 564 |
| FM-1 | 156690*2 | 135424*2 | 86.43 | 135424 | 134964 | 99.66 | 252/1 | 106276 | 497 |
| FM-2 | 173087*2 | 167177*2 | 96.59 | 167177 | 166821 | 99.79 | 252/1 | 123318 | 598 |
| FM-3 | 178823*2 | 166624*2 | 93.18 | 166624 | 166174 | 99.73 | 252/1 | 112869 | 540 |
| FM-4 | 180667*2 | 168741*2 | 93.40 | 168741 | 168309 | 99.74 | 252/1 | 114406 | 701 |

P50, Palbio 50 RD, a dried porcine solubles; S50, Soyppt-50%, a enzyme-treated soybean meal; CDCP, concentrated degossypolized cottonseed protein; FM, fish meal.

**Supplementary table S2.** Number of observed species, richness and diversity indices in the caecal samples from each sample.

| Sample Name | Observed species | Chao | Ace | Shannon | Simpson | Good’s coverage |
| --- | --- | --- | --- | --- | --- | --- |
| P50-1 | 354 | 425.15 | 426.07 | 3.26 | 0.09 | 0.9994 |
| P50-2 | 565 | 602.82 | 611.69 | 4.27 | 0.03 | 0.9995 |
| P50-3 | 623 | 682.38 | 688.44 | 4.39 | 0.03 | 0.9993 |
| P50-4 | 560 | 639.47 | 648.21 | 4.24 | 0.03 | 0.9993 |
| P50-5 | 569 | 627.52 | 634.66 | 4.40 | 0.03 | 0.9993 |
| S50-1 | 295 | 335.53 | 350.32 | 2.88 | 0.13 | 0.9996 |
| S50-2 | 742 | 830.39 | 809.98 | 4.86 | 0.02 | 0.9991 |
| S50-3 | 606 | 663.06 | 658.27 | 4.35 | 0.03 | 0.9994 |
| S50-4 | 697 | 777.50 | 764.25 | 4.58 | 0.02 | 0.9993 |
| S50-5 | 648 | 741.19 | 733.45 | 4.16 | 0.04 | 0.9991 |
| CDCP-1 | 725 | 820.64 | 810.53 | 4.54 | 0.03 | 0.9992 |
| CDCP-2 | 712 | 775.02 | 770.94 | 4.74 | 0.02 | 0.9992 |
| CDCP-3 | 623 | 686.79 | 694.80 | 4.15 | 0.04 | 0.9992 |
| CDCP-4 | 579 | 682.47 | 651.35 | 3.74 | 0.06 | 0.9992 |
| CDCP-5 | 564 | 683.28 | 654.46 | 3.03 | 0.20 | 0.9991 |
| FM-1 | 497 | 588.74 | 557.27 | 4.02 | 0.03 | 0.9992 |
| FM-2 | 598 | 705.35 | 694.95 | 3.63 | 0.08 | 0.9991 |
| FM-3 | 540 | 614.82 | 613.50 | 4.08 | 0.04 | 0.9992 |
| FM-4 | 701 | 794.64 | 787.33 | 4.36 | 0.03 | 0.9990 |

P50, Palbio 50 RD, a dried porcine solubles; S50, Soyppt-50%, a enzyme-treated soybean meal; CDCP, concentrated degossypolized cottonseed protein; FM, fish meal.
